# Improving the measurement resolution in BOTDR sensor with optimized wavelet denoising strategy

**DOI:** 10.1371/journal.pone.0341131

**Published:** 2026-01-21

**Authors:** Mustafa Essa Hamzah, Mohd Saiful Dzulkefly Zan, Abdulwahhab Essa Hamzah, Mohd Faisal Ibrahim, Ahmad Ashrif A. Bakar, Hisham Mohamad, Yosuke Tanaka

**Affiliations:** 1 Department of Electrical, Electronic and Systems Engineering, Faculty of Engineering and Built Environment, Universiti Kebangsaan Malaysia (UKM), Bangi, Selangor, Malaysia; 2 Department of Communications Engineering, Iraq University College, Basrah, Iraq; 3 Civil & Environmental Engineering Department, Universiti Teknologi PETRONAS, Seri Iskandar, Perak, Malaysia; 4 Division of Advanced Electrical and Electronic Engineering, Institute of Engineering, Tokyo University of Agriculture and Technology, Naka-cho, Koganei, Tokyo, Japan; University of Baghdad, IRAQ

## Abstract

Signal averaging imposes a significant trade-off between measurement time and measurand resolution in a sub-meter differential cross-spectrum Brillouin optical time domain reflectometry (DCS-BOTDR). This article introduces an optimized post-processing strategy that integrates wavelet denoising (WD) function with a traditional Lorentzian curve fitting (LCF) under relatively low signal averages to mitigate this problem. Symlet, Daubechies, Coiflet, and Biorthogonal Spline WD functions with a 4-level decomposition were executed on a six-core CPU utilizing a single-program-multiple-data (SPMD) paradigm and compared. Experimental validation was executed over distances of 350 meters and 1.21 kilometers of single-mode fiber. The experimental results demonstrate a 2.7 times reduction in required signal averages, in which the proposed LCF + WD method achieved a 2.7 MHz Brillouin frequency shift (BFS) resolution with only 21000 averages, a performance that required 56000 averages using the LCF method alone. To manage the computational load on the large experimental datasets, the algorithm was implemented on a parallel six-core architecture, accelerating the data processing speed by up to 4.8 times compared to serial computation. The method also successfully preserved a 0.4 m spatial resolution and improved temperature resolution to 3°C across a 1.21 km fiber at just 14000 signal averages. In comparison to other methods such as machine learning-based enhancements, the proposed strategy presents a more straightforward, training-free execution that attains comparable BFS and temperature resolutions without the necessity of extensive datasets or rigorous model training. Together with the multicore architecture, the proposed strategy is particularly beneficial for real-time distributed sensing applications where computational resources may be constrained.

## 1. Introduction

In recent years, Brillouin-based distributed fiber optic sensors have become a powerful technology for monitoring strain and temperature along tens of kilometers of fiber [1]. Brillouin optical time domain reflectometry (BOTDR) is one of the well-known Brillouin-based distributed fiber optic sensors that have been studied for years [2]. Unlike point-based optical sensing techniques [3,4], BOTDR offers a wider sensing range with excellent measurement distribution and sensing performance. BOTDR generates spontaneous Brillouin scattering (SpBS) in fiber from the interaction between a pulsed probe and an acoustic wave [5]. The frequency difference between the pulsed probe and the generated SpBS is known as the Brillouin frequency shift (BFS). BFS exhibits a linear relationship with the change in temperature and strain along the fiber, making it valuable for distributed temperature and strain monitoring [6]. BOTDR is capable of measuring strain and temperature in a distributed manner, making them advantageous for monitoring large sensing areas. The applications include structural health monitoring of civil infrastructures and leakage detection along oil and gas pipelines [7–10].

To estimate a BFS from a measured Brillouin gain spectrum (BGS), the Lorentzian curve fitting (LCF) function is predominantly used due to the Lorentzian shape of a BGS. However, LCF is time-consuming and prone to significant errors, particularly when the signal-to-noise ratio (SNR) is low. A sharp spectral profile can be achieved from a strong Brillouin gain interaction, low noise level, or improved SNR [11]. However, in the case of BOTDR, due to the nature of the SpBS, the amplitude of the generated Brillouin signal is small, and this results in the difficulty in achieving high BFS resolution, as well as high spatial resolution.

To improve the spatial resolution of BOTDR to sub-meter order, several techniques such as double-pulse BOTDR (DP-BOTDR) [12], differential cross spectrum BOTDR (DCS-BOTDR) [13], phase shift pulse BOTDR (PSP-BOTDR) [14] and differential BOTDR [15] have been proposed. However, the techniques reported in [13–15] suffer lower SNR measurement than the conventional single pulse BOTDR despite the high spatial resolution attainment, due to the pulse differential calculation in the signal processing stage that removes the SpBS signal contributed by the long pulses; this consequently degrades the BFS resolution. The BFS resolution measurement from the above methods can be improved by increasing the signal averaging, which however requires longer measurement and signal processing times.

Alternatively, encoding the optical pulse of the BOTDR with certain codes such as Golay complementary sequences [16,17], or modulating several pulses with various phase modulation schemes [18] have improved the SNR and at the same time attain the sub-meter spatial resolution. Despite the additional number of pulses significantly improving the BFS resolution, these techniques add more complex encoding and decoding processes that further require more computational resources and consequently longer processing time.

In addition, machine learning (ML)-based methods have been proposed for further improving the BFS resolution and minimizing the requirements for averaging for faster processing time. Artificial neural networks (ANN) [19,20], and support vector machines (SVM) [21] have exhibited considerable enhancements in the accuracy of BFS estimation, particularly in the presence of noisy environmental conditions. However, these models necessitate substantial training datasets, complex parameter tuning, and considerable computational resources to attain a level of generalization. Furthermore, the necessity for retraining often arises when parameters (such as fiber type, temperature ranges, or laser linewidth) undergo alterations, thereby rendering ML methods less feasible for real-time applications in BOTDR.

Thus, in this article, we propose the deployment of a simple yet effective wavelet denoising (WD) method after the LCF calculation of the DCS-BOTDR signals, alongside parallel computing for improving the BFS resolution and the signal processing speed under relatively low signal averages. Through experimental analysis across test fiber lengths of 350 m and 1.21 km, the capability of parallel processing in improving the signal processing speed was successfully demonstrated. Utilizing six CPU cores, the system processed 1750 Brillouin spectra over a 350 m fiber and 6050 spectra over a 1.21 km fiber, achieving a 4.5 ~ 4.8 times faster speed improvement compared to serial computation. With just 21000 signal averages, the LCF + WD approach attained 2.7 MHz of BFS resolution along the 350 m fiber, which is comparable to that of the conventional LCF averaged 56000 times. This results in an overall 7.5 times the increase in processing efficiency. With the LCF + WD method, we also observed an enhancement in the temperature resolution as accurate as 3°C along the 1.21 km test fiber at just 14000 signal averages, with the maximum magnitude of temperature resolution enhancement of 4.3°C. The method also preserved a sub-meter spatial resolution of 0.4 m, confirming its capability in enhancing the quality of noisy DCS-BOTDR signals. By overcoming the limitations of conventional processing techniques, the LCF + WD method provides a simple but highly efficient and precise BFS measurement in a BOTDR sensor.

## 2. Brillouin spectrum calculation using FFT-based differential cross-correlation

**Fig 1:**
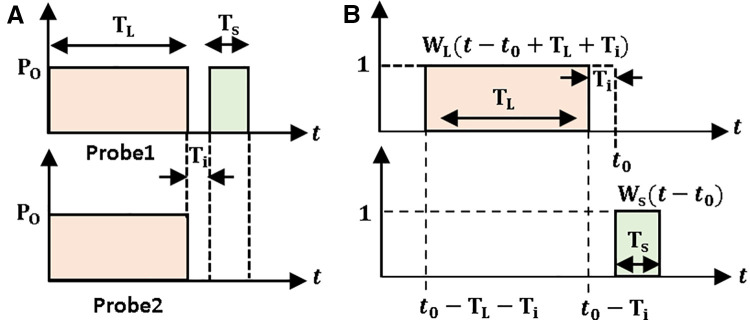
(a) The pair of pulsed probes used in DCS-BOTDR. **(b)** Two window functions to sample the backscattered light measured by each probe.

In a DCS-BOTDR technique, an intensity modulation configuration is used to produce a pair of probe pulses, as shown in Fig 1 (a). The first probe (Probe 1) contains a pulse of long-period *T*_*L*_ and another one of short-period *T*_*S*_, separated by an interval *T*_*i*_. The second probe (Probe 2) only contains a pulse of the same long period *T*_*L*_. The signal processing begins by sampling the Brillouin signals measured by both pulsed probes. During sampling, the periods of the narrow and wide window samplings are set to *T*_*S*_ and *T*_*L*_, respectively, as illustrated in Fig 1 (b) [22].

The analysis is followed by cross correlating the data obtained from the wide and narrow window samplings. The data $b_{L}^{x}\left(t,t_{\text{0}}\right)$ sampled by the wide window function and $b_{S}^{x}\left(t,t_{\text{0}}\right)$ by the narrow window function are obtained by integrating the backscattered signal along the fiber at a distance *z*, which are given by [22]


$$b_{L}^{x}\left(t,t_{\text{0}}\right)=\int_{\text{0}}^{\infty }\tilde{b_{L}^{x}}\left(t,t_{\text{0}},z^{\prime }\right)dz^{\prime }$$ (1)

$$b_{S}^{x}\left(t,t_{\text{0}}\right)=\int_{\text{0}}^{\infty }\tilde{b_{S}^{x}}\left(t,t_{\text{0}},z^{\prime \prime }\right)dz^{\prime \prime }$$ (2)

where the superscript *x* is either 1 or 2, i.e., Probe 1 or Probe 2, $\tilde{b_{L}^{x}}\left(t,t_{\text{0}},z^{\prime }\right)$and $\tilde{b_{S}^{x}}\left(t,t_{\text{0}},z^{\prime }\right)$are respectively the backscattered light signals in the time domain measured by the long probe- and the short probe pulses.

Finally, the Brillouin spectra for a short fiber section is obtained by applying FFT on the produced signal from differential cross-correlation between Probe 1 and Probe 2 signals, as shown below:


$$\left|\left\langle FFT\left[b_{L}^{\text{1}}\left(t,t_{\text{0}}\right)\right]\cdot {FFT\left[b_{S}^{\text{1}}\left(t,t_{\text{0}}\right)\right]}^{*}\right\rangle -\left\langle FFT\left[b_{L}^{\text{2}}\left(t,t_{\text{0}}\right)\right]\cdot {\;FFT\left[b_{S}^{\text{2}}\left(t,t_{\text{0}}\right)\right]}^{*}\right\rangle \right|$$ (3)

The product in Eq. (3) determines the bandwidth of the resultant spectrum, which is influenced by the pulse of long-duration *T*_*L*_ and phonon lifetime, while the signal differential gives the spatial resolution determined by *T*_*S*_ [22].

## 3. Parallel processing based on multicore processor

Efficient signal processing in high-resolution BOTDR systems necessitates the management of extensive volumes of BGS data, particularly when each dataset encompasses thousands of time-domain traces and FFT-derived spectra. To mitigate the limitations associated with serial computation, multicore parallel processing emerges as a viable solution by allocating computational workloads across multiple cores within a single processor chip [23]. A multicore processor incorporates several autonomous processing units (cores) to facilitate the simultaneous execution of instructions [24]. Each core functions with its own arithmetic and logic unit (ALU), control unit, and cache memory, while sharing higher-level memory and input/output buses. Multicore processors confer enhanced data exchange rates and reduced latency among cores, rendering them particularly suitable for high-throughput scientific computations.

In the present investigation, all computational tasks were executed on an Intel® Core™ i7-9750H CPU operating at 2.60 GHz, comprising six physical cores and twelve logical threads. The processing algorithm was formulated and executed within the MATLAB R2022a Parallel Computing Toolbox, which offers inherent support for distributed and multicore processing through various models, including parfor, SPMD, and batch. Among these models, the single SPMD model was chosen for its adaptability in managing extensive numerical arrays derived from BOTDR measurements.

**Fig 2:**
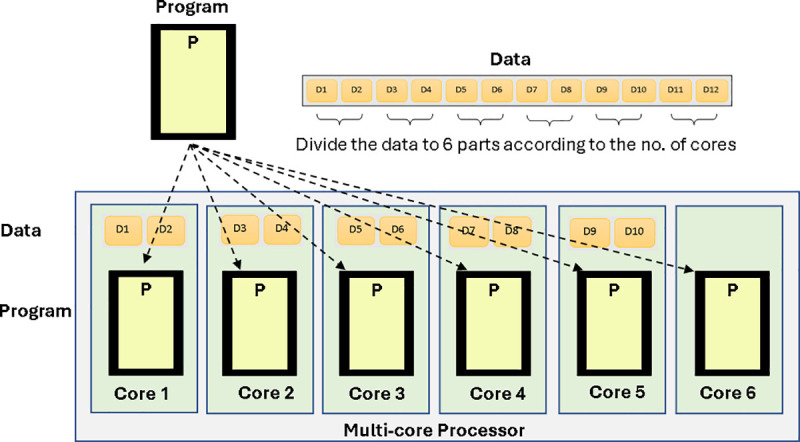
SPMD execution model.

The SPMD framework permits multiple cores to concurrently execute identical program code, with each core processing a unique segment of the dataset [25]. As depicted in Fig 2, each core undertakes the processing of a subset of the input BGS data corresponding to designated fiber segments, while synchronization among cores transpires via shared memory or message passing. This architectural design guarantees that FFT-based differential cross-correlation, LCF, and WD operations are conducted independently across data partitions. Upon the completion of their designated computations by all cores, the results are collated and combined into a singular output array that represents the comprehensive BFS distribution along the fiber.

The implementation workflow initiates with the equitable division of the total number of captured Brillouin traces among the six cores. For example, within the context of the 350 m fiber test comprising 1750 spectra, each core was responsible for processing approximately 292 spectra. Each core executed FFT transformation, BGS construction, and LCF fitting locally. Subsequently, the sym4-based WD procedure was executed in parallel to attenuate high-frequency noise while preserving the predominant BFS features. This architectural configuration minimized inter-core data transfer, thereby achieving near-linear scaling efficiency.

The deployment of MATLAB’s parallel pool with six cores yielded a 4.5 ~ 4.8 times reduction in computational time relative to serial execution. The total execution duration for 1750 spectra was reduced from 496 minutes to approximately 102 minutes for full-resolution analysis, with results are consistent with those obtained through serial processing. Furthermore, the SPMD model facilitates reproducibility and scalability, by which augmenting the number of available cores or executing the same MATLAB script on a computing cluster would proportionately enhance processing speed without necessitating algorithmic alterations. The incorporation of SPMD-based multicore parallelization within MATLAB’s Parallel Computing Toolbox thus exhibits reliable scalability, reproducibility, and compatibility with real-time BOTDR data acquisition systems, which is also applicable for integration with other methodologies such as adaptive filtering and machine learning.

## 4. Wavelet Denoising (WD)

Wavelet denoising (WD) technique has been studied for its potential in improving the signal accuracy and while preserving the essential signal characteristics [26,27]. The WD method is employed to denoise the measured signal, ensuring it remains non-disruptive under severe environmental conditions. In our analysis, WD is implemented after modifying LCF to reduce noise, improve SNR, and enhance the BFS resolution. The main feature of WD technique that makes it suitable for the DCS-BOTDR is that it can isolate and suppress high-frequency noise while retaining low-frequency features. WD would also preserve the sub-meter spatial performance of the DCS-BOTDR. Besides, it is also computationally efficient and suitable for real-time or parallel processing. WD operates by decomposing a signal into its frequency components using wavelet transforms, effectively separating the signal into various scales and frequencies. This method allows for multi-resolution control and detailed information retrieval in both the time and frequency domains. Consequently, time windows can be adjusted for different signals and analysis states [27]. The process involves the following three steps:

1) Decomposition: The original signal is decomposed into different frequency components or scales using a wavelet transform. This transform highlights both high and low-frequency components, which helps in identifying noise.
2) Thresholding: After decomposition, the noise-containing components are identified and suppressed by applying a thresholding technique. Thresholding involves setting small coefficients (considered as noise) to zero or reducing their magnitude significantly. The universal threshold, λ

$$\lambda =\sigma \sqrt{\text{2}logN}$$ (4)

was deployed, where σ is the noise standard deviation and *N* is the data number.

3) Reconstruction: The denoised signal is reconstructed by utilizing the modified coefficients from which noise has been suppressed. This reconstructed signal aims to retain essential features of the original signal while removing unwanted noise.

A one-dimensional signal can be represented by [26]


    S(k)=f(k)+εe(k) (5)

where *k* = 0,1,2,3…*n*‒1, S(k denotes the raw signal, f(k the actual signal with noise, and e(k) the Gaussian noise with amplitude coefficient of ε. In Eq. (5), f(k) consists of the low-frequency signals, while e(k the high-frequency ones. Denoising is computed to reduce the high-frequency noise and reconstruct the low-frequency signal; f(k) is the signal approximation that needs to be optimized, while εe(k) is to be reduced.

## 5. Experimental setup

**Fig 3:**
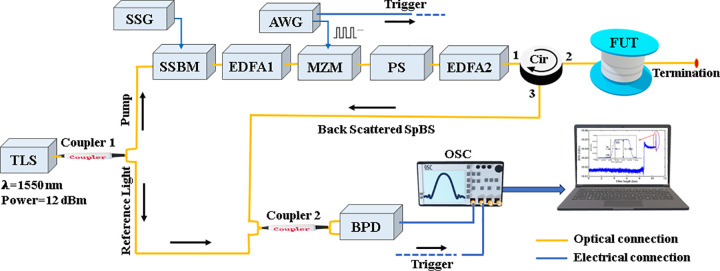
The DCS-BOTDR experimental setup. (TLS: Tunable laser source, SSG: Synthesized signal generator, SSBM: Single side band modulator, EDFA: Erbium doped fiber amplifier, MZM: Mach-Zehnder modulator, AWG: arbitrary waveform generator, BPD: Balance photodetector, FUT: fiber under test, OSC: Oscilloscope).

The experimental setup of the DCS-BOTDR is shown in Fig 3. Initially, a tunable laser source (TLS) generates continuous wave (CW) light at a wavelength of 1550 nm and an output power of 12 dBm. A 1 × 2 coupler (coupler 1) splits the optical power equally into two arms. The bottom arm coupled the light from the TLS directly to a 2 × 2 coupler (coupler 2) as the reference light for the heterodyne detection. The top arm is connected to a single sideband modulator (SSBM) for frequency modulation. At the SSBM, the CW light’s frequency is upshifted by approximately 10.08 GHz, with the electrical signal for frequency modulation is supplied by a synthesized signal generator (SSG). The frequency-shifted light is then amplified by an Erbium-doped fiber amplifier (EDFA1) achieving about −8 dBm (~0.2 mW) of output power, before being modulated in its amplitude by a Mach-Zehnder modulator (MZM) into an optical pulse. An arbitrary waveform generator (AWG) was used to produce an electrical pulse to the MZM for the modulation.

To mitigate fading noise due to polarization, a polarization scrambler (PS) is employed. Further amplification of the probe pulse is achieved using an additional EDFA (EDFA2). The optical pulse power injected into the fiber via an optical circulator (Cir) was around 300 mW, which was sufficient to generate the SpBS along the test fiber. In our experiment, the Stokes component of the SpBS was extracted from the test fiber for analysis. The SpBS signal beats with the reference light before being detected by a balanced photodetector (BPD). This signal is digitized by an oscilloscope (OSC) at the sampling speed of 5 GS/s and then processed by a personal computer.

**Fig 4:**
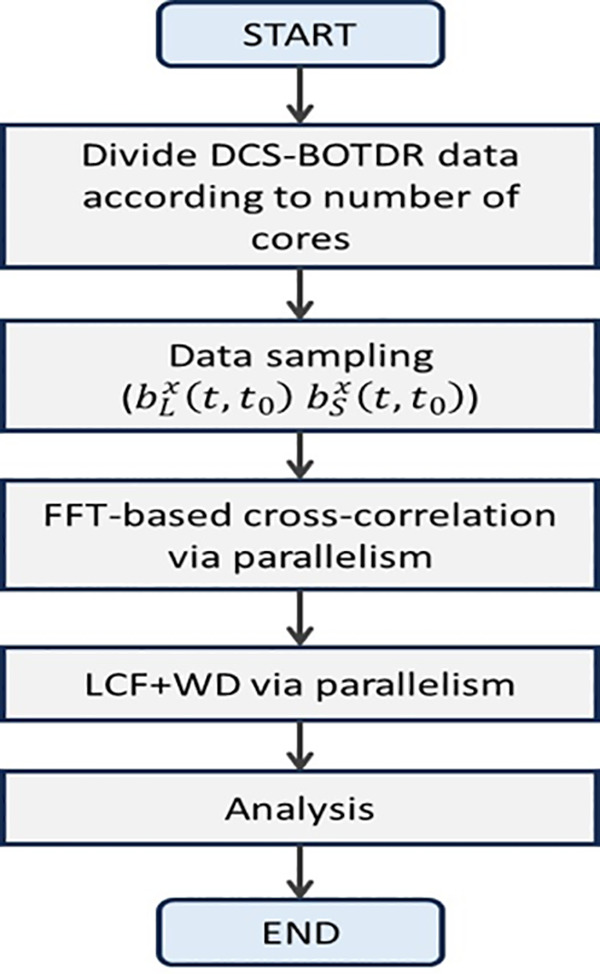
Flowchart of the BGS and BFS calculations via LCF + WD method accelerated by the parallel processing.

Fig 4 presents the flowchart of the BGS and BFS calculations incorporated with LCF + WD method through parallel processing. Furst, the captured DCS-BOTDR time domain traces will be divided equally according to the CPU cores. Then, the data will be sampled for the FFT calculations explained in Section 2 via parallel processing to produce the Brillouin spectra. These spectra will be further processed by LCF + WD method, also through parallelism before being analyzed for final data analysis.

## 6. Results and analysis

**Fig 5:**
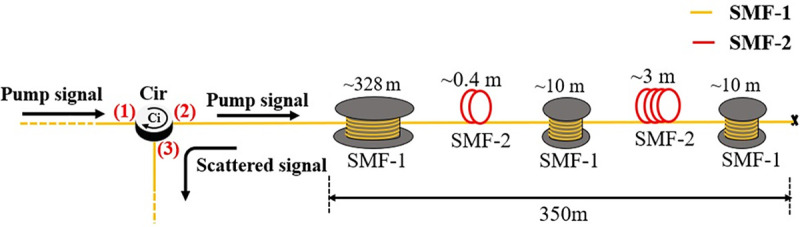
Configuration of the 350 m fiber under test (FUT).

Several experiments have been conducted by measuring 350 m and 1.21 km test fibers (both were the standard ITU-T G.652.D telecommunication fibers) for analyzing the performance of the DCS-BOTDR sensor based on the proposed WD filtering and parallel processing methods. In the first experiment, as shown in Fig 5, a 350 m FUT was fabricated by splicing two single mode fibers (SMF) having different BFS values for simulating temperature difference between both fibers. The BFS difference was also implemented to validate the BFS accuracy by the proposed WD method. In detail, the BFS for SMF-1 was about 10.90 GHz while SMF-2 10.85 about GHz, which results in about 50 MHz difference. Considering the Brillouin temperature coefficient of 1.01 MHz/°C [28,29], this translates into about 50°C temperature different. To evaluate the spatial resolution, the 350 m FUT was segmented into five alternating sections of SMF-1 and SMF-2. The five sections consist of the following configuration: a 328 m section of SMF-1, a 0.4 m section of SMF-2, followed by a 10 m section of SMF-1, a 3 m section of SMF-2, and finally another 10 m section of SMF-1.

The beat signal detected by a balanced photodetector (BPD) was sampled 56000 times and analyzed by the OSC before being processed by using the proposed method. The sampling point in each DCS-BOTDR time domain trace for the FFT and LCF + WD calculations was fixed to 2 ns (equivalent to 0.2 m spatial point), which translates into 1750 number of time domain data for the 350 m test fiber case. The pulsed probe input power was set to about 300 mW by EDFA2 in Fig 3. Several durations of $T_{L}$ were utilized, specifically 2 ns, 4 ns, 10 ns, 14 ns, 20 ns, and 24 ns, while the interval $T_{i}$ and short pulse duration$\;T_{s}$ were fixed at 0.5 ns and 2 ns, respectively. The spatial resolution obtained from the experiment was expected to be around 0.2 m. For the parallel processing technique, the data segmentation per core was based on the SPMD method, considering the PC’s CPU consisting of six processors (Intel (R) Core (TM) i7-9750H CPU@2.60 GHz, 6 Cores, 12 Logical Processors). Subsequently, 56000 traces were divided per CPU core, for which a significant reduction in execution time is expected.

### 6.1. Signal processing by the conventional LCF method

**Fig 6:**
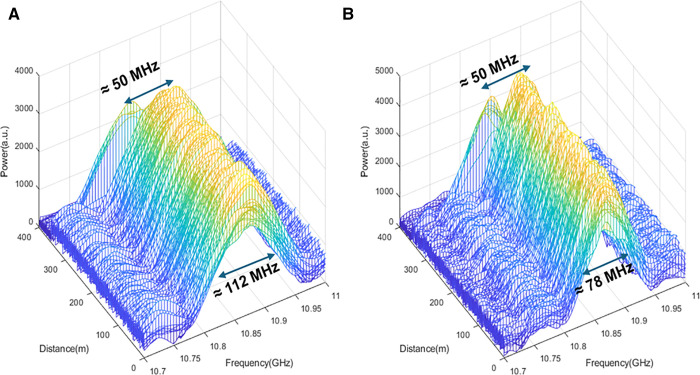
Brillouin spectra along the 350 m FUT processed by the conventional LCF method for *T*_*L*_ of (a) 10 ns, and (b) 14 ns cases. **(a)** *T*_*L*_ = 10 ns **(b)** *T*_*L*_ = 14 ns.

To analyze the effectiveness of LCF + WD method in the DCS-BOTDR, we started by analyzing the experimental results of the DCS-BOTDR signals processed by the conventional LCF method alone. Figs 6 (a) and (b) respectively illustrate the representative of 1750 Brillouin spectra along the 350 m test fiber measured by $T_{L}\;$ of 10 ns and 14 ns cases. For $T_{L}$ = 10 ns case, the Brillouin spectrum width analyzed at the full-width-at-half-maximum (FWHM) was around 112 MHz, while for $T_{L}$= 14 ns case, the spectrum width has narrowed to about 78 MHz, confirming the inverse proportionality of the spectrum width with $T_{L}$.

**Fig 7:**
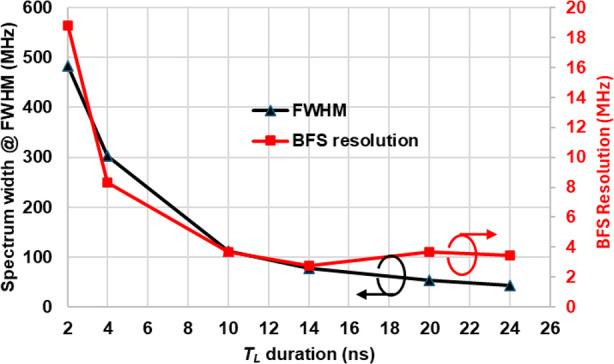
Effect of the pulse duration ${𝐓}_{L}$ on the Brillouin spectrum width and BFS resolution along the 350 m fiber, processed by the conventional LCF method.

Fig 7 (left y-axis) illustrates the effect of $T_{L}$ on the spectrum width. As $T_{L}$ increases from 2 ns to 24 ns, the spectrum width has narrowed from 483.3 MHz to 43.9 MHz. Fig 7 (right y-axis) also shows the change in the BFS resolution (calculated by standard deviation) with $T_{L}$. For $T_{L}$ from 2 ns to 24 ns, the BFS resolution has improved from about 19 MHz to 2.7 MHz. For all $T_{L}$ cases, the signal processing time via conventional serial calculation took about 496 minutes (~8 hours) to calculate the FFT-cross correlation for all 1750 BGS after 56000 times of averaging, with each spectrum having 1024 datapoints. Thus, on average, it took about 17 seconds to calculate a BGS through the serial processing.

From these results, we will demonstrate later the effectiveness of the proposed LCF + WD method and the parallel processing in improving the BFS resolution and the processing time of the DCS-BOTDR at lower than 56000 signal averages, while preserving its sub-meter spatial resolution feature. Performance parameters such as pulse duration ($T_{L}$), number of averages, and fiber length were accounted for in the analysis. It should be noted that although the representative results shown here pertain to short fiber, in general, the effect of the pulse duration on the spectrum width and the BFS resolution is generally similar for any fiber length.

### 6.2. Incorporating the LCF with WD method accelerated by the parallel processing

To identify the optimal wavelet for improving Brillouin frequency shift (BFS) resolution, we performed a comparative analysis of four wavelet functions: Symlet (sym4), Daubechies (db4), Coiflet (coif4), and Biorthogonal Spline (bior4.4). Each function, using 4-level decomposition, was applied to the DCS-BOTDR signals measured along the 350 m test fiber by *T*_*L*_ = 14 ns case. To accelerate the calculation, six-core parallel processing was deployed.

**Fig 8:**
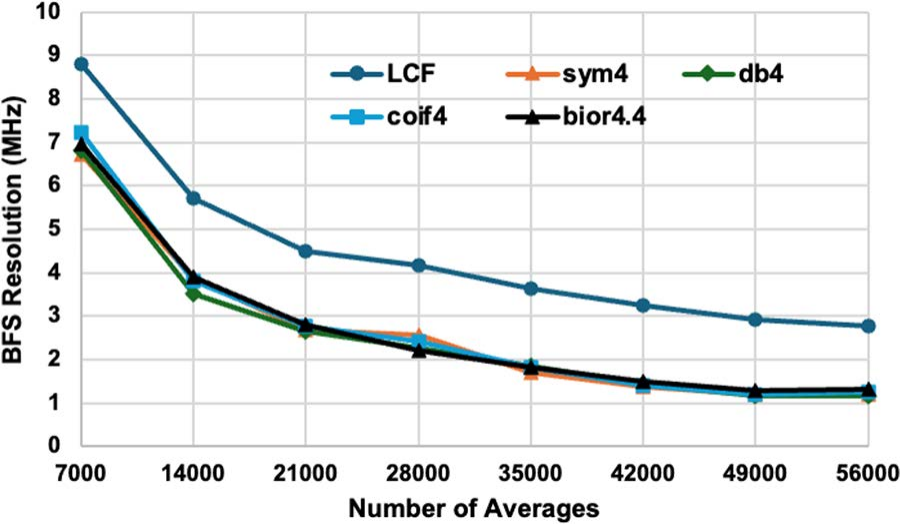
Compare effect of adding different types of wavelet functions sym4, db4, coif4 and bior4.4 on the BFS resolution at different averaging times for ${𝐓}_{L}$=14 ns case. The test fiber length was 350 m.


The BFS resolution for each WD function was statistically evaluated by calculating the standard deviation of the BFS data between the 250 m and 300 m locations, as described in Eq. (6),


$$BFS\;resolution=\sqrt{\frac{\text{1}}{N}\sum_{i=\text{1}}^{N}{\left(y-\overset{―}{y}\right)}^{\text{2}}},$$ (6)

where *y* is the measured value of BFS, $\overset{―}{y}$ the average of the estimated BFS and *N* the quantity of data. The standard deviation, i.e., the BFS resolution results obtained by the WD functions were then compared with the conventional LCF method across various signal averages, as shown in Fig 8. The conventional LCF approach established a baseline performance, with its BFS resolution improving from 8.8 MHz at 7000 averages to 2.7 MHz at 56000 averages. In contrast, all WD methods delivered a substantial improvement, achieving resolutions below 3 MHz at 21000 averages and higher. Additionally, the analysis on the measurement accuracy was also conducted. In detail, the root means square error (RMSE) was calculated for all WD cases and the conventional LCF using the Eq. (7).

$$RMSE=\sqrt{\frac{\text{1}}{N}\sum_{i=\text{1}}^{N}{\left(y-\hat{y}\right)}^{\text{2}},}$$ (7)

where $\hat{y}$ is the true BFS value. The true BFS value was obtained from the temperature of the fiber measured by a thermocouple, before being transformed into a BFS value using the associated Brillouin temperature coefficient of 1.01 MHz/°C. Taking the BFS values from the 56000 times average case, the RMSE for all WD functions and the conventional LCF are shown in Table 1. It was observed that all WD functions gave better RMSE than the conventional LCF method, and almost similar with the standard deviation results for 56000 times average depicted in Fig 8, indicating the consistency of the resolution (standard deviation) and the accuracy (RMSE) calculated by all WD functions.

**Table 1:** Root-mean-square error (RMSE) for all WD functions and conventional LCF under the case of 56000 times average.

Wavelet function	RMSE (MHz)
sym4	1.47
db4	1.45
coif4	1.46
bior4.4	1.46
conventional LCF	3.10

As observed in Table 1 and Fig 8, all four WD functions consistently produced almost similar BFS resolutions and accuracy (RMSE) across all averaging conditions. However, considering the near-symmetrical structure and compact support features of sym4 that provides an optimal balance between effective noise removal and the preservation of the Brillouin signals, we chose this wavelet function for all further analysis.

The resultant BFS resolution achieved by the LCF + WD (sym4) approach ranged from 6.73 MHz at low averaging to 1.19 MHz at high averaging. This reflects an improvement in BFS resolution of approximately 2 MHz compared to the conventional LCF method. Although increasing the number of averages improves the BFS resolution, it concurrently increases the measurement time. Importantly, the inclusion of sym4 function substantially improves resolution even at low number of averages. Notably, while the conventional LCF method required 56000 averages to achieve a BFS resolution of 2.7 MHz, the LCF + WD method attained the same resolution with only 21000 averages, representing a 2.7 times reduction in processing time.

**Fig 9:**
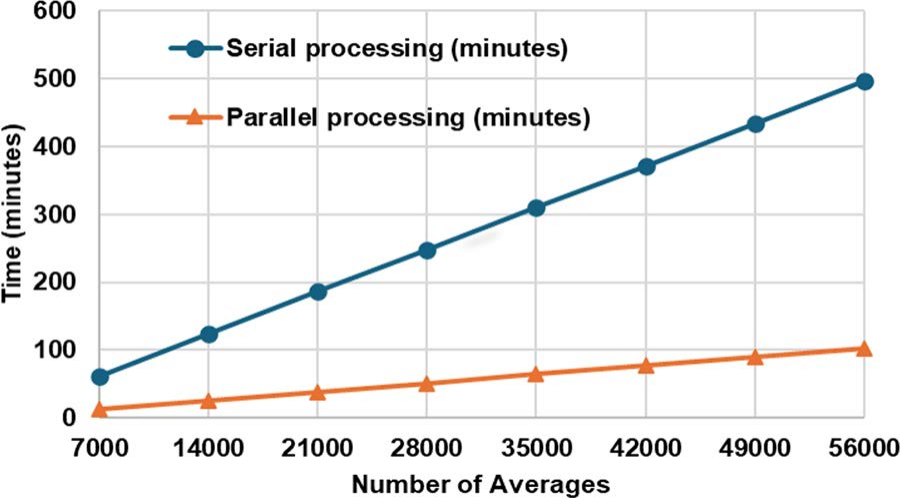
The effect of parallel processing on the processing time in comparison with the serial processing in producing 1750 Brillouin spectra along the 350 m test fiber.

Fig 9 illustrates the effect of signal averaging on the processing time for generating a total of 1750 Brillouin spectra along the 350 m fiber, for $T_{L}=$ 14 ns case. The graph compares the processing time by serial processing (in blue) and six-cores parallel processing (in orange), with both showing a linear increase with the number of averages. For serial processing, the FFT calculation time ranges from 62 to 496 minutes. In contrast, parallel processing with six cores significantly reduces the time to between 13 and 102 minutes, achieving nearly 4.8 times faster processing.

**Fig 10:**
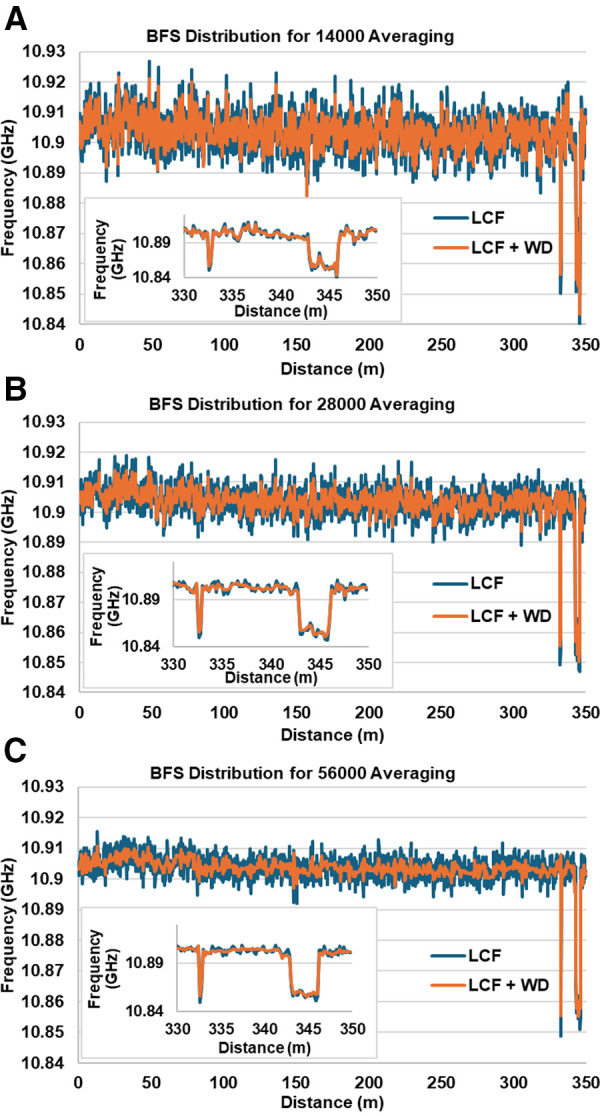
BFS distribution comparison between that obtained by the conventional LCF method and that by the LCF + WD (sym4) method under (a) 14000, (b) 28000 and (c) 56000 times averaging. The inset Fig each shows the BFS difference between SMF-1 and SMF-2 cables.

To verify the robustness of the LCF + WD method in preserving the feature of the DCS-BOTDR signal and the enhancement in the BFS resolution along the 350 m test fiber, a BFS distribution comparison was made between the conventional LCF method and that of the LCF + WD (sym4) method for $T_{L}=$ 14 ns case. As representative, Figs 10 (a), (b) and (c) show the comparison of BFS distribution between the LCF + WD method and the conventional LCF method 14000, 28000 and 56000 signal averages respectively.

For all averaging conditions, the LCF + WD method produces smaller BFS fluctuation than the conventional LCF, indicating an improved BFS resolution measurement. In addition, as observed in each Fig, the LCF + WD method still preserves the main feature of the original DCS-BOTDR signal. In detail, from the inset Figs in Fig 10, the BFS measurement at SMF-1 and SMF-2 cables has been distinguished clearly. Furthermore, as explained by Fig 9, the deployment of six cores parallel processing in the LCF + WD method has sped up the calculation time by a factor of 4.8. As discussed previously, by referring to the results illustrated in the Figs 8 and 9, the 21000 times of averaging (38 minutes processing time) for the LCF + WD method produced the same BFS resolution of 2.7 MHz as that by 56000 times (102 minutes) for the conventional LCF method, resulting in about 2.7 times faster processing speed. Thus, combining this with the effect of parallel processing, the total processing time was 7.5 times faster.

**Fig 11:**
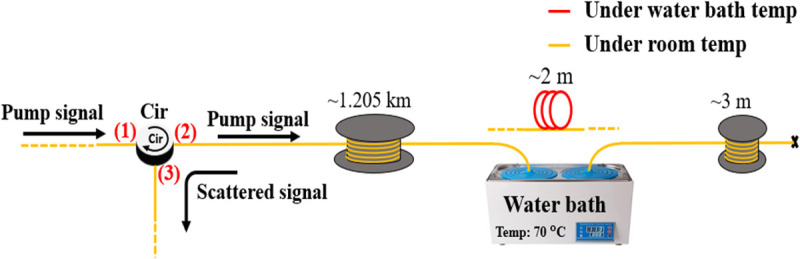
Configuration of the temperature measurement along the 1.21 km test fiber.

**Fig 12:**
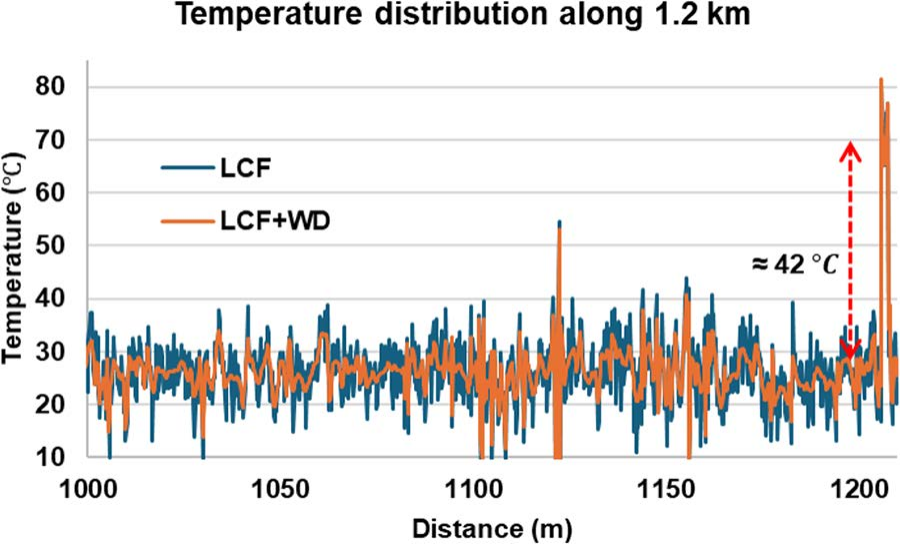
Temperature distribution along the 1.21 km fiber for both conventional LCF and LCF + WD methods after 14000 times average (*T*_*L*_ = 18 ns case). At the far end of the fiber, a 2 m section was heated to demonstrate the shift in the BFS.

To evaluate the effectiveness of the LCF + WD method and parallel processing under various pulse durations, we measured a 1.21 km test fiber with $T_{L}=$ 6, 10, 14, 18, 22 and 30 ns pulses, while the $T_{i}$ and $T_{s}$ were fixed at 2 and 4 ns, respectively. The pulse input power was set to 600 mW. The time domain signals were captured at only 14000 times for averaging purposes. Fig 11 shows the arrangement of the 1.21 km test fiber for the experiment, with a 2 m section at the far end heated to approximately 70°C using a water bath equipment. The experiment was conducted at a room temperature of 27°C. As a representative, Fig 12 illustrates the BFS distribution along the 1.21 km fiber for the *T*_*L*_ = 18 ns case, where a 2 m heated section at the far end of the fiber was observed. It is confirmed that the deployment of the LCF + WD method has improved the BFS resolution measurement compared to the conventional LCF method. The BFS resolution processed by the conventional LCF- and LCF + WD methods was 4.9°C and 3°C respectively. Despite the low number of averages of only 14000 times for measuring the 1.21 km fiber, a 1.9°C temperature resolution enhancement has been successfully demonstrated by the LCF + WD method. At location 1122 m, there was a slight fluctuation in the temperature, which was due to the imperfection in the fiber setup and thus could be ignored in the analysis.

Fig 12 also shows the fiber-heated location, which exhibited an approximate temperature increase of 42°C. Considering the Brillouin temperature coefficient of approximately 1.01 MHz/°C [28,29], the BFS corresponding to the room temperature (27°C) was around 10.860 GHz. From this relationship, the 42.9 MHz frequency shift translates into about 42°C [30], confirming the correct measurement of temperature change at the 2 m heated section at the far end of the fiber. In terms of processing time, for generating 6050 Brillouin spectra along the 1.21 km fiber, with each spectrum containing 1024 datapoints and processed at 14000 signal averages, it took approximately 440 minutes using serial processing and 98 minutes using parallel processing. Thus, it required approximately 4.4 seconds to generate a Brillouin spectrum with serial processing and only about 0.97 seconds with parallel processing. This indicates that the parallel process has accelerated 4.5 times faster processing, nearly as expected. We then analyzed the rising and falling edges of the signal at the 2 m heated section to confirm the robustness of the LCF + WD method in preserving the spatial resolution of the DCS-BOTDR. As a result, a 0.4 m spatial resolution measurement was observed for both the conventional LCF and the proposed LCF + WD methods, corresponding to $T_{s}=\;$ 4 ns.

**Fig 13:**
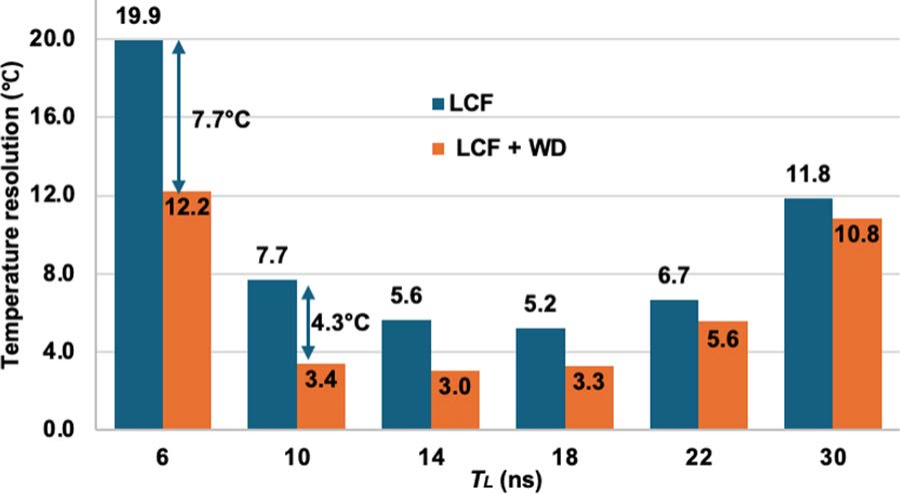
Comparison of BFS resolution enhancement between the conventional LCF method and LCF + WD method for different ${𝐓}_{L}$ durations after 14000 times average for the 1.21 km fiber.

Fig 13 compares the temperature resolution between the conventional LCF method and the proposed LCF + WD for $T_{L}$ range values from 6 to 30 ns for the 1.21 km fiber. The relationship between $T_{L}$ and the temperature resolution agrees well with the previous report on the DCS-BOTDR technique [22], confirming the consistency of the DCS-BOTDR technique in achieving optimum measurand resolution for *T*_*L*_ between 10 ns and 22 ns. In the case of conventional LCF, for $T_{L}$ between 6 ns and 30 ns, the highest temperature resolution was 5.2°C ($T_{L}=$18 ns), while the lowest 19.9°C ($T_{L}=\;$6 ns). However, with the LCF + WD method, the temperature resolution (i.e., the BFS resolution) has been significantly enhanced. For the same $T_{L}$ range, the temperature resolution has improved to a range between 3°C ($T_{L}=$14 ns) and 12.2°C ($T_{L}=\;$6 ns). Even though the temperature resolution enhancement was the highest for *T*_*L*_ = 6 ns case (7.7°C), the resultant temperature resolution of 12.2°C after the deployment of LCF + WD was still much poorer than that of *T*_*L*_ = 10, 14, 18 and 22 ns cases. The use of *T*_*L*_ duration of shorter than the phonon lifetime (~10 ns) had generated very low SNR of Brillouin signal, which had consequently resulted in a poor temperature resolution. Thus, from Fig 13, it can be observed that the LCF + WD has further enhanced the temperature resolution of the DCS-BOTDR for the optimum *T*_*L*_ of between 10 and 22 ns, confirming the effectiveness of the proposed method in refining the noisy data obtained from the DCS-BOTDR at low signal averages.

**Fig 14:**
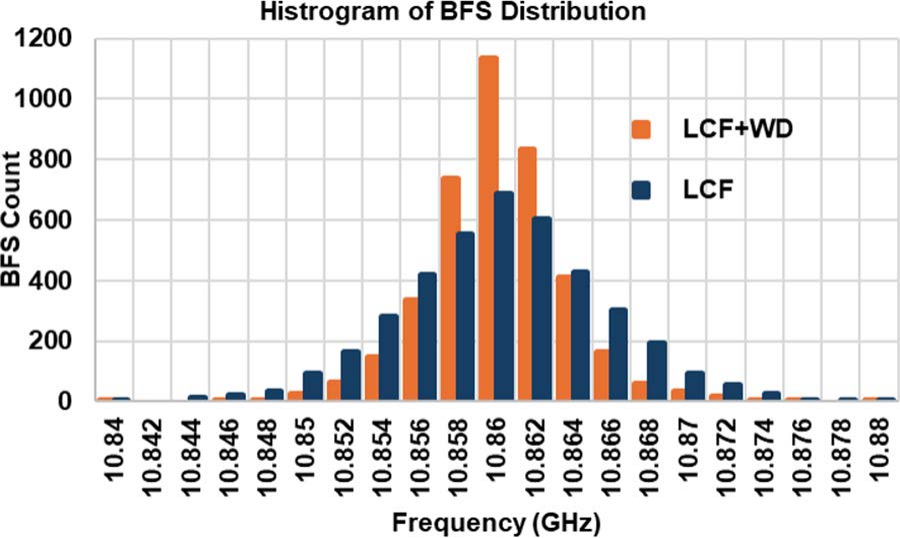
Histogram of BFS distribution along the 1.21 km fiber for both LCF + WD and LCF methods in the case of $T_{L}=$ 18 ns.

To further validate the BFS resolution enhancement by the LCF + WD method, we provide the comparative statistical analysis of the BFS distribution between the LCF + WD and the conventional LCF methods. As a representative, Fig 14 illustrates the histogram of 4000 BFS distribution data along the 1.21 km fiber for both methods in the case of *T*_*L*_ = 18 ns. In terms of the mean value, both methods gave similar mean of BFS of 10.860 GHz. However, the standard deviation of the BFS, i.e., the BFS resolution for the LCF + WD is around 3.6 MHz, which is better than that of the conventional LCF of 5.4 MHz, as expected. This also resulted in a smaller margin error of 0.11 MHz compared to 0.17 MHz for the conventional LCF. Considering a 95% confidence interval, the range of BFS distribution processed by the LCD + WD method was between 10.86009 GHz and 10.86032 GHz, which was narrower than that for the conventional LCF method of between 10.86003 GHz and 10.86036 GHz. The significant reduction in the BFS distribution’s width and in the BFS deviations concludes the efficacy of the WD method in attenuating high-frequency noise prior to the LCF process, thereby yielding a more accurate and reliable BFS estimation.

## 7. Comparative analysis of computational complexity and scalability

To evaluate the applicability of the proposed LCF + WD methodology, its computational attributes were compared with the traditional LCF processing and the ML-based enhancement algorithms for Brillouin-based sensors [19–21]. From a computational perspective, the LCF method requires sufficient numbers of data in spectral points within each Brillouin gain spectrum (BGS) for BFS estimation, and this also represents the needs of iterative fitting operations. By integrating WD into the LCF method, the overall process requires a modest escalation to attributable to the wavelet transform operations. Nevertheless, the noise attenuation effect diminishes the required iterations for convergence in the LCF, thereby equilibrating the total computational requirements. Moreover, the implementation of parallel processing allocates the computation across six CPU cores, providing almost a linear scalability and realizing a practical speedup factor ranging from 4.5 to 4.8 times without compromising the accuracy of BFS estimation.

Conversely, ML-based techniques such as artificial neural networks (ANN) or support vector machines (SVM), embody a significant training complexity, typically affected by the number of network layers, number of neurons and training epochs. ML techniques necessitate substantial data labeling for training and validation to attain model generalization under fluctuating noise and environmental conditions. Upon completion of the training process, ML models can deduce BFS with minimal latency; however, retraining or transfer learning is frequently imperative when there are alterations in measurement range, experimental configurations and more importantly different fiber type. Different fiber cable has different BFS at room temperature, and thus this results in further complexity in data training in ML techniques. This challenge of adaptability escalates maintenance requirements and constrains scalability across diverse fiber configurations.

In contrast, the LCF + WD methodology offers a deterministic strategy characterized by uniform performance across a spectrum of fiber lengths and measurement conditions. Its computational burden scales linearly with the dataset size and can be seamlessly distributed across multicore or GPU architectures. As a result, the proposed approach presents a more balanced factor between algorithmic simplicity, processing efficiency, and scalability, rendering its appropriateness for real-time or large-scale distributed fiber sensing applications.

## 8. Conclusion

In summary, this investigation has elucidated an advanced signal processing methodology for DCS-BOTDR systems through the incorporation of wavelet denoising (WD) with Lorentzian curve fitting (LCF), further expedited by multicore parallel processing. The proposed LCF + WD significantly diminishes signal averaging necessities while maintaining sub-meter spatial resolution and enhancing the Brillouin frequency shift (BFS) and consequently the temperature resolution. In addition to the enhancement demonstrated by a 2.7 times reduction in number of averages and a 4.8 times acceleration in computational speed, this approach signifies a remarkable advancement toward real-time, high-precision distributed fiber sensing.

Crucially, this research underscores a transition from solely hardware-centric improvements to computationally efficient signal processing techniques. The synergy of WD and parallel computing offers a versatile framework that can be seamlessly integrated into the existing BOTDR systems without necessitating hardware alterations. This merging framework also establishes a scalable foundation for next-generation Brillouin-based distributed fiber sensing, characterized by increased speed, intelligence, and suitability for the sophisticated monitoring systems anticipated in the future. In future, the incorporation of this framework with artificial intelligence (AI) and embedded edge computing platforms may facilitate autonomous, adaptive noise mitigation and instantaneous data interpretation. Such integration would amplify the relevance of BOTDR technology for extensive smart infrastructure applications, encompassing structural health monitoring, energy asset management, and environmental sensing networks.

## Supporting information

S1 Data Raw Data. (XLSX)
